# Coronavirus peplomer interaction

**DOI:** 10.1063/5.0120167

**Published:** 2022-11-30

**Authors:** Myong Chol Pak, R. Chakraborty, M. A. Kanso, K. Tontiwattanakul, Kwang-Il Kim, A. J. Giacomin

**Affiliations:** 1 Department of Physics, Kim Il Sung University, Taesong District, Pyongyang 999093, Democratic People's Republic of Korea; 2 Chemical Engineering Department, Jadavpur University, Kolkata 700032, India; 3 Chemical Engineering Department, Polymers Research Group, Queen's University, Kingston, Ontario K7L 3N6, Canada; 4 Department of Mechanical and Aerospace Engineering, King Mongkut's University of Technology North Bangkok, Bangkok, Thailand; 5 Mechanical and Materials Engineering Department, Queen's University, Kingston, Ontario K7L 3N6, Canada; 6 Physics, Engineering Physics and Astronomy Department, Queen's University, Kingston, Ontario K7L 3N6, Canada; 7 Mechanical Engineering Department, University of Nevada, Reno, Nevada 89557-0312, USA

## Abstract

By virtue of their lack of motility, viruses rely entirely on their own temperature (Brownian motion) to position themselves properly for cell attachment. Spiked viruses use one or more spikes (called peplomers) to attach. The coronavirus uses adjacent peplomer pairs. These peplomers, identically charged, repel one another over the surface of their convex capsids to form beautiful polyhedra. We identify the edges of these polyhedra with the most important peplomer hydrodynamic interactions. These convex capsids may or may not be spherical, and their peplomer population declines with infection time. These peplomers are short, equidimensional, and bulbous with triangular bulbs. In this short paper, we explore the interactions between nearby peplomer bulbs. By interactions, we mean the hydrodynamic interferences between the velocity profiles caused by the drag of the suspending fluid when the virus rotates. We find that these peplomer hydrodynamic interactions raise rotational diffusivity of the virus, and thus affect its ability to infect.

## I. INTRODUCTION

By virtue of their lack of motility, viruses rely entirely on their own temperature (Brownian motion) to position themselves properly for cell attachment.^1^ For such attachment, the virus faces the Goldilocks problem: (i) too little rotational diffusivity and the alignment time will exceed the chemical attachment kinetics requirement and (ii) too much rotational diffusivity and the alignment time will subceed this kinetics requirement.^2,3^ Spiked viruses use one or more spikes (called *peplomers*) to attach (Sec. I of Ref. 1). The coronavirus uses adjacent peplomer pairs. These peplomers, identically charged, repel one another over the surface of their convex capsids to form beautiful polyhedra.^4,5^ These convex capsids may or may not be spherical,^6^ and their peplomer population declines after infection.^7^ These peplomers are short, equidimensional, and bulbous with triangular bulbs.^8^ In this short paper, we approximate the spike hydrodynamics with those of a single sphere (see Sec. VII of Ref. 1).

To deepen our understanding of viruses, or the suspension of any particles of complex shape for that matter, we can replace the particle with a rigid bead-rod structure. The beads represent sites of local drag following Stokes flow. The (nonexistent) dimensionless and massless rods represent the rigidly fixed distances between adjacent or nearby beads. In this concise paper, we consider only the hydrodynamic interferences between nearby beads. By *nearby*, we mean beads whose centers terminate a common edge of the polyhedral solution to the Thomson problem (see this justified on p. 113101-2 of Sec. I in Ref. 13). We thus consider only spherical capsids, leaving aspherical ones for another day.

**FIG. 1.**
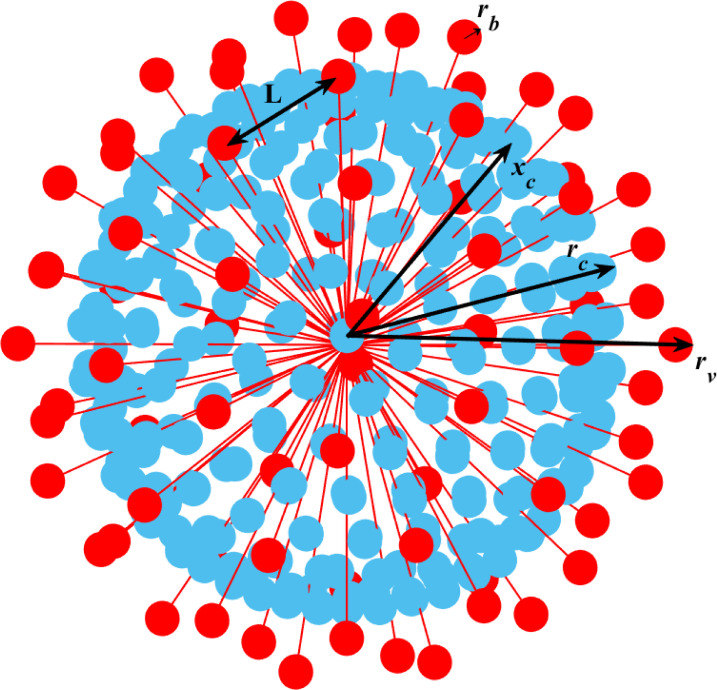
The model of coronavirus.

In this short paper, we explore the interactions between nearby peplomer bulbs. By *interactions*, we mean the hydrodynamic interferences between the velocity profiles caused by the drag of the suspending fluid when the virus rotates. We focus specifically on the average number of peplomers on a coronavirus particle immediately after infection,^4,9,10^ that is, $N_{p}=74$ (see Fig. 5 of Ref. 1). Tables I (with Fig. 1) and II define our symbols and variables, respectively, dimensional and dimensionless. Though our work is mainly driven by curiosity, its public health implications have not escaped our attention. For instance, we know that the relevant mutations differentiating successive variants of SARS-CoV-2 are mutations affecting the peplomer proteins.^11^ How these peplomer mutations affect their shapes, sizes, or populations is however not known.

**TABLE I.** Dimensional variables. *M*: mass; *L*: length; *t*: time.

Name	Unit	Symbol
Angular frequency	$t^{-1}$	ω
Bead diameter	L	d
Bead mass	M/bead	m
Bead friction coefficient	M/t	ζ
Bead radius (Fig. 1)	L	$r_{b}\equiv d/2$
Capsid radius (Fig. 1)	L	$r_{c}$
Characteristic length (Fig. 1)	L	L
Energy values in molecular-scale systems	$ML^{2}/t^{2}$	kT
Minus imaginary part of complex viscosity (modified)	M/Lt	${\tilde{\eta }}^{\prime \prime }$
Moments of inertia (modified)	$ML^{2}$	${\tilde{I}}_{1},{\;\tilde{I}}_{2},\;{\;\tilde{I}}_{3}$
Peplomer bulb radius	L	$r_{p}$
Position vector of the *i*th bead and *j*th element with respect to the center of mass	L	$R_{ij}$
Radius difference, capsid minus bead (Fig. 1)	L	$x_{c}\equiv r_{c}-r_{b}$
Real part of complex viscosity (modified)	M/Lt	${\tilde{\eta }}^{\prime }$
Relaxation time of rigid dumbbell	t	$\lambda_{0}\equiv \pi d\eta_{s}L^{2}/4kT$
Relaxation time of suspension	t	λ
Relaxation time of suspension (modified)	t	$\tilde{\lambda }$
Rotational diffusivity (modified)	$t^{-1}$	${\tilde{D}}_{r}$
Solvent viscosity	M/Lt	$\eta_{s}$
Temperature	T	T
Time	t	t
Virus radius (Fig. 1)	L	$r_{v}\equiv \;r_{p}+r_{b}$
Viscosity, zero-shear (modified)	M/Lt	${\tilde{\eta }}_{0}$
Zero-shear first normal stress difference	M/L	$\Psi_{1,0}$

**TABLE II.** Dimensionless variables and groups. *M*: mass; *L*: length; *t*: time.

Name	Symbol
Hydrodynamic interaction parameter [Eq. (6)]	A≡d/2L
Modified coefficients in the expression for the shear relaxation function in columns 5–7 of Table IV	$\tilde{a},\;\tilde{b},\;\tilde{\nu }$
Modified lopsidedness in column 8 of Table IV	$\frac{2\tilde{b}}{\tilde{a}\tilde{\nu }}$
Total number of beads	N
Total number of peplomers	$N_{p}$
Total number of capsid beads	$N_{c}$
Characteristic bead-rod length	$\ell \equiv L/x_{c}$

For general rigid bead-rod theory with hydrodynamic interaction, for the real and *minus* the imaginary parts of the coronavirus contribution to the complex viscosity, we get [Eqs. (23) and (24) of Ref. 12]

$$\frac{{\tilde{\eta }}^{\prime }\left(\tilde{\lambda }\omega \right)-\eta_{s}}{{\tilde{\eta }}_{0}-\eta_{s}}={\left(\frac{1}{2\tilde{b}/\tilde{a}\tilde{\nu }}+1\right)}^{-1}\left(\frac{1}{2\tilde{b}/\tilde{a}\tilde{\nu }}+\frac{1}{1+{\left(\tilde{\lambda }\omega \right)}^{2}}\right)$$ (1)

and

$$\frac{{\tilde{\eta }}^{\prime \prime }\left(\tilde{\lambda }\omega \right)}{{\tilde{\eta }}_{0}-\eta_{s}}={\left(\frac{1}{2\tilde{b}/\tilde{a}\tilde{\nu }}+1\right)}^{-1}\frac{\tilde{\lambda }\omega }{1+{\left(\tilde{\lambda }\omega \right)}^{2}},$$ (2)

where the tildes signify with hydrodynamic interaction, namely, where A>0.^10,12^ The best way to get $\tilde{\lambda }$ is to fit Eq. (1) to experimental observations at low frequency ( $\tilde{\lambda }\omega <1$), though, for the coronavirus, we know of no such data.

From the fitted values of $\tilde{\lambda }$, we get the dimensionless rotational diffusivity of the virus from [see Eqs. (22) and (23) of Ref. 1]

$$\lambda_{0}{\tilde{D}}_{r}=\lambda_{0}\frac{1}{6\tilde{\lambda }}=\frac{\tilde{\nu }}{72},$$ (3)

which we will use below.

**FIG. 2.**
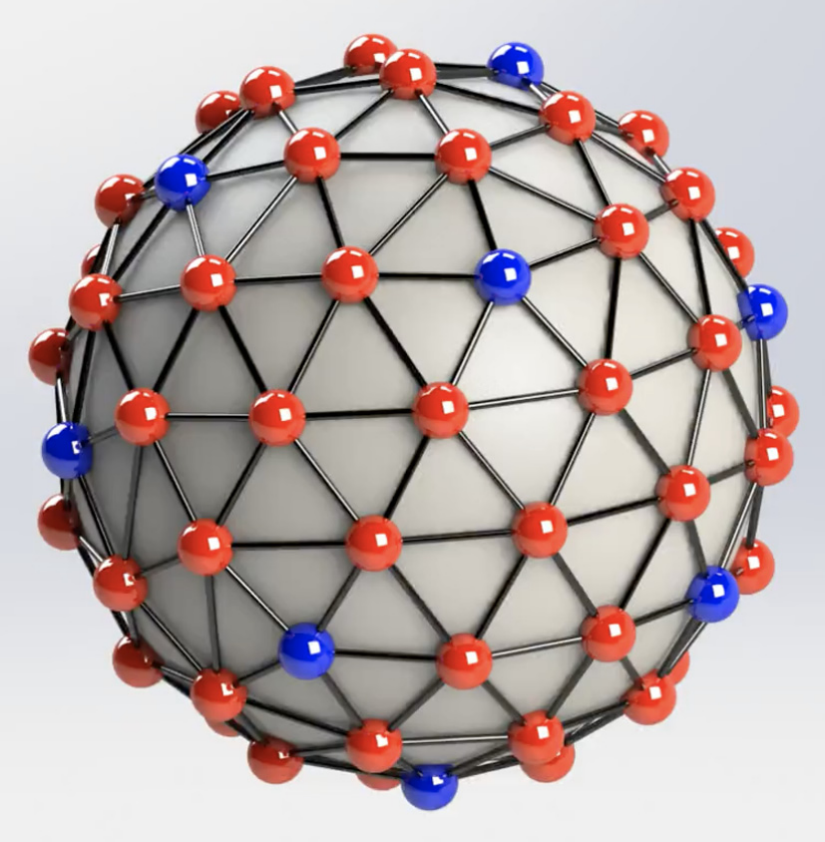
Hydrodynamic interaction between peplomers sharing common edge of polyhedral solution to Thomson problem. The peplomer population $\left(N_{p}=74\right)$ arranges itself on the 74 vertices of this 144-face, and 216-edge polyhedron. Multimedia view: https://doi.org/10.1063/5.0120167.1
10.1063/5.0120167.1

We find that for $N_{p}=74$, 12 peplomers form just five polyhedral edges, and 62 form six edges. We count 216 edges on our 74-vertex peplomer polyhedron, which Fig. 2 (Multimedia view) illustrates and animates. We further find that these nearby peplomer hydrodynamic interactions raise the rotational diffusivity of the virus, and thus affect its ability to infect.

General rigid bead-rod theory distinguishes itself from theories for a suspension of spheres, about which general rigid bead-rod theory is silent. General rigid bead-rod theory, after all, arrives at the complex viscosity through the orientation distribution function in small-amplitude oscillatory shear flow.^13^ By contrast, spheres in suspension are without orientation.

## II. METHOD

In this paper, we employ the recent method of Secs. II of Refs. 8 and 14, and specifically, we neglect “other” terms in Eqs. (29)–(30) and (18)–(19), respectively. By *other*, we mean peplomer hydrodynamic interactions other than those between ends of each polyhedral edge.

**FIG. 3.**
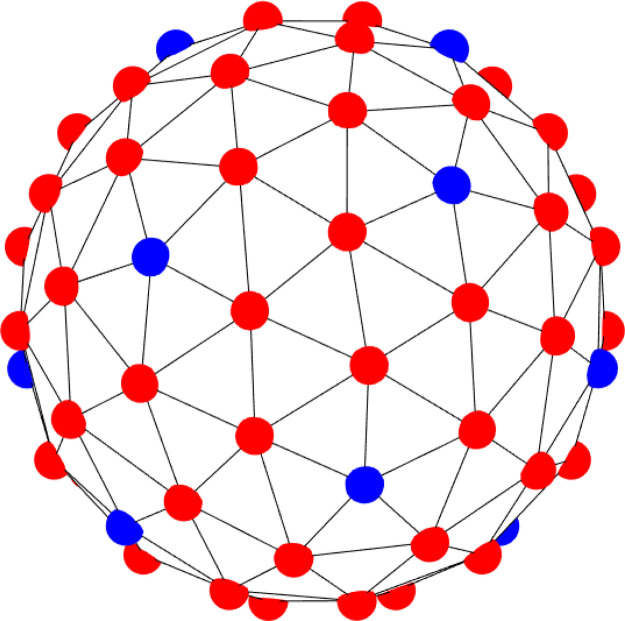
Polyhedron with 12 peplomers (blue) having five nearby neighbors, and 62 peplomers (red) having six.

Figure 3 illustrates the structure of the peplomer population $N_{p}=74$ spread over the surface of the capsid. We find 12 peplomers (blue in Fig. 3) with five nearby neighbors, and 62 peplomers (red in Fig. 3) with six. In other words, the peplomer population arranges itself onto 74 vertices of the 144-sided polyhedron, with 216 edges. Our Thomson solutions of course satisfy Euler's theorem, V−E+F=2 (where V=74, E=216, and F=144). We tabulate, in Cartesian coordinates, the peplomer positions for $N_{p}=74$. Peplomer positions marked in blue in Table III have just five nearby neighbors, and the rest, six. On average, each peplomer has 216/37 nearby neighbors.

**TABLE III.** The structure of peplomers in coronavirus ( $N_{c}=256,\;N_{p}=74,\;r_{v}/r_{c}=5/4$).

Bead number	$R_{\nu 1}$	$R_{\nu 2}$	$R_{\nu 3}$	Count of the nearest beads	Number of nearest beads
1	0.167 044	−1.193 66	0.331 328	6	9, 11, 28, 33, 40, 47
2	−0.654 55	−1.040 44	0.227 051	5	6, 28, 33, 52, 62
3	−0.204 84	−0.177 53	1.220 267	6	13, 16, 22, 49, 65, 70
4	0.654 546	1.040 444	0.227 051	5	18, 39, 56, 67, 71
5	0.672 641	−0.593 12	0.870 791	6	11, 20, 27, 40, 61, 70
6	−1.026 93	−0.704 76	0.105 964	6	2, 37, 38, 46, 52, 62
7	−0.672 64	0.593 123	0.870 791	6	16, 22, 29, 35, 50, 51
8	0.524 298	0.807 227	−0.797 48	6	19, 26, 32, 45, 66, 71
9	0.676 317	−1.050 59	0.036 82	6	1, 27, 40, 42, 47, 68
10	0.331 665	0.674 009	0.999 115	6	22, 39, 50, 5, 6, 65, 74
11	0.162 602	−0.913 2	0.837 944	6	1, 5, 13, 33, 40, 70
12	−1.209 42	0.158 896	0.273 078	6	35, 37, 38, 51, 57, 73
13	−0.331 67	−0.674 01	0.999 115	6	3, 11, 33, 49, 52, 70
14	−1.082 08	−0.073 19	−0.621 47	6	15, 17, 21, 37, 46, 57
15	−0.775 6	−0.389 9	−0.899 39	5	14, 17, 30, 36, 46
16	−0.574 94	0.135 271	1.101 667	5	3, 7, 22, 35, 49
17	−0.711 85	0.128 377	−1.019 45	6	14, 15, 21, 30, 43, 69
18	0.237 441	1.227 239	0.002 569	6	4, 24, 32, 56, 59, 71
19	0.065 255	0.700 595	−1.033 14	6	8, 26, 32, 43, 60, 69
20	0.989 257	−0.146 63	0.749 924	6	5, 27, 48, 61, 63, 74
21	−0.946 67	0.473 954	−0.664 58	6	14, 17, 57, 58, 69, 73
22	−0.185 36	0.488 073	1.135 759	6	3, 7, 10, 16, 50, 65
23	0.501 273	−0.610 81	−0.968 57	6	25, 31, 42, 54, 55, 64
24	−0.167 04	1.193 661	0.331 328	6	18, 29, 41, 50, 56, 59
25	0.235 966	−0.991 61	−0.723 54	5	23, 31, 42, 44, 47
26	0.343 392	0.277 63	−1.169 39	6	8, 19, 43, 45, 55, 64
27	1.001 383	−0.621 94	0.415 848	6	5, 9, 20, 40, 63, 68
28	−0.237 44	−1.227 24	0.002 569	6	1, 2, 33, 44, 47, 62
29	−0.598 71	0.962 491	0.526 945	5	7, 24, 41, 50, 51
30	−0.343 39	−0.277 63	−1.169 39	6	15, 17, 31, 36, 43, 64
31	−0.065 25	−0.700 59	−1.033 14	6	23, 25, 30, 36, 44, 64
32	0.219 548	1.115 249	−0.520 1	6	8, 18, 19, 59, 60, 71
33	−0.287 81	−1.056 07	0.603 659	6	1, 2, 11, 13, 28, 52
34	1.082 081	0.073 189	−0.621 47	6	45, 53, 54, 55, 66, 72
35	−0.989 26	0.146 629	0.749 924	6	7, 12, 16, 38, 49, 51
36	−0.524 3	−0.807 23	−0.797 48	6	15, 30, 31, 44, 46, 62
37	−1.219 69	−0.261 71	−0.079 75	6	6, 12, 14, 38, 46, 57
38	−1.098 73	−0.332 87	0.494 471	6	6, 12, 35, 37, 49, 52
39	0.768 62	0.746 169	0.644 18	6	4, 10, 48, 56, 67, 74
40	0.598 708	−0.962 49	0.526 945	5	1, 5, 9, 11, 27
41	−0.676 32	1.050 59	0.036 82	6	24, 29, 51, 58, 59, 73
42	0.702 464	−0.907 75	−0.495 01	6	9, 23, 25, 47, 54, 68
43	−0.189 94	0.230 767	−1.213 73	6	17, 19, 26, 30, 64, 69
44	−0.219 55	−1.115 25	−0.520 1	6	25, 28, 31, 36, 47, 62
45	0.775 595	0.389 903	−0.899 39	5	8, 26, 34, 55, 66
46	−1.000 64	−0.598 05	−0.451 16	6	6, 14, 15, 36, 37, 62
47	0.258 696	−1.201 86	−0.226 04	6	1, 9, 25, 28, 42, 44
48	1.098 728	0.332 874	0.494 471	6	20, 39, 53, 63, 67, 74
49	−0.746 96	−0.327 6	0.947 235	6	3, 13, 16, 35, 38, 52
50	−0.162 6	0.913 197	0.837 944	6	7, 10, 22, 24, 29, 56
51	−1.001 38	0.621 942	0.415 848	6	7, 12, 29, 35, 41, 73
52	−0.768 62	−0.746 17	0.644 18	6	2, 6, 13, 33, 38, 49
53	1.219 692	0.261 707	−0.079 75	6	34, 48, 63, 66, 67, 72
54	0.946 67	−0.473 95	−0.664 58	6	23, 34, 42, 55, 68, 72
55	0.711 848	−0.128 38	−1.019 45	6	23, 26, 34, 45, 54, 64
56	0.287 809	1.056 066	0.603 659	6	4, 10, 18, 24, 39, 50
57	−1.200 78	0.252 514	−0.238 45	5	12, 14, 21, 37, 73
58	−0.702 46	0.907 746	−0.495 01	6	21, 41, 59, 60, 69, 73
59	−0.258 7	1.201 865	−0.226 04	6	18, 24, 32, 41, 58, 60
60	−0.235 97	0.991 614	−0.723 54	5	19, 32, 58, 59, 69
61	0.574 939	−0.135 27	1.101 667	5	5, 20, 65, 70, 74
62	−0.699 91	−0.990 99	−0.300 93	6	2, 6, 28, 36, 44, 46
63	1.209 416	−0.158 9	0.273 078	6	20, 27, 48, 53, 68, 72
64	0.189 944	−0.230 77	−1.213 73	6	23, 26, 30, 31, 43, 55
65	0.204 84	0.177 527	1.220 267	6	3, 10, 22, 61, 70, 74
66	1.000 639	0.598 05	−0.45116	6	8, 34, 45, 53, 67, 71
67	1.026 933	0.704 757	0.105 964	6	4, 39, 48, 53, 66, 71
68	1.030 085	−0.700 66	−0.102 44	6	9, 27, 42, 54, 63, 72
69	−0.501 27	0.610 808	−0.968 57	6	17, 19, 21, 43, 58, 60
70	0.185 364	−0.488 07	1.135 759	6	3, 5, 11, 13, 61, 65
71	0.699 909	0.990 992	−0.300 93	6	4, 8, 18, 32, 66, 67
72	1.200 78	−0.252 51	−0.238 45	5	34, 53, 54, 63, 68
73	−1.030 09	0.700 663	−0.102 44	6	12, 21, 41, 51, 57, 58
74	0.746 958	0.327 598	0.947 235	6	10, 20, 39, 48, 61, 65

From Table II, we learn that A≡d/2L, namely, that the extent of the hydrodynamic interaction between peplomers is dimensionless, and just depends on peplomer geometry. Specifically, it depends upon peplomer size and separation. Combining the dimensionless length

$$\ell \equiv L/x_{c}$$ (4)

and

$$r_{c}=x_{c}+r_{b}$$ (5)

with Eq. (5) of Ref. 9

$$A\equiv \frac{d}{2L}=\frac{2r_{b}}{2x_{c}\ell }=\frac{r_{b}}{(r_{c}-r_{b})\ell }=\frac{1}{(r_{c}/r_{b}-1)\ell }$$ (6)

and mindful of Eq. (69) of Ref. 1, we get

$$\frac{35}{2}\leq \frac{r_{c}}{r_{b}}\leq \frac{41}{2}$$ (7)

and l=0.5529. The physical dimensions of peplomers thus dictate the following range for peplomer hydrodynamic interaction:

9.28≤100A≤10.96. (8)

Therefore, throughout this work we parametrize peplomer hydrodynamic interaction as A=0.09, 0.10, 0.11.

The L entering Eq. (6) is the center-to-center distance between nearest neighbors. This separation will of course differ from one peplomer population, $N_{p}$, to another. As a consequence, the range Eq. (8) may or may not apply when $N_{p}$ differs significantly from 74. This short paper just focuses on $N_{p}=74$, namely, the average peplomer population immediately after infection. In previous work, which neglects hydrodynamic interaction, we set d=L arbitrarily. Otherwise put, so long as hydrodynamic interaction is neglected we can osculate beads (see Table IX of Ref. 1). However, when incorporating hydrodynamic interaction, we must set $L=0.5529x_{c}$ ( l=0.5529), as we have done herein.

## III. RESULTS

**FIG. 4.**
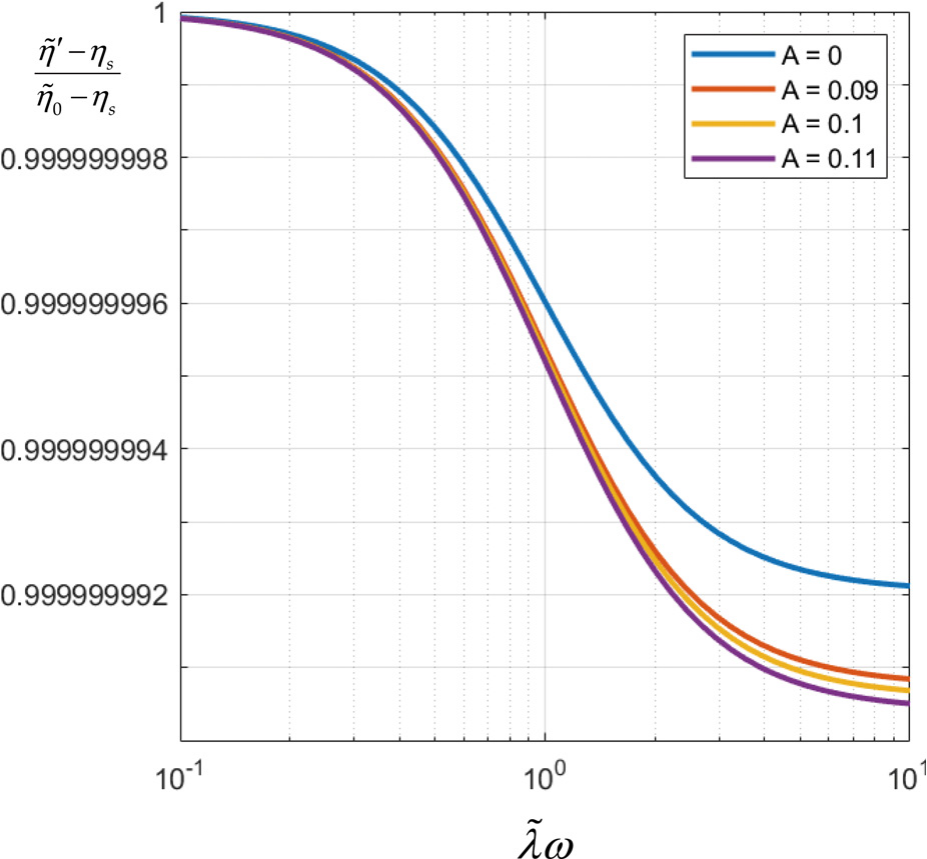
Dimensionless normalized $\eta^{\prime }\left(\lambda \omega \right)$ from Eq. (1) parametrized with hydrodynamic interaction parameter, A, over the physical range arrived at for the coronavirus, Eq. (8).

**FIG. 5.**
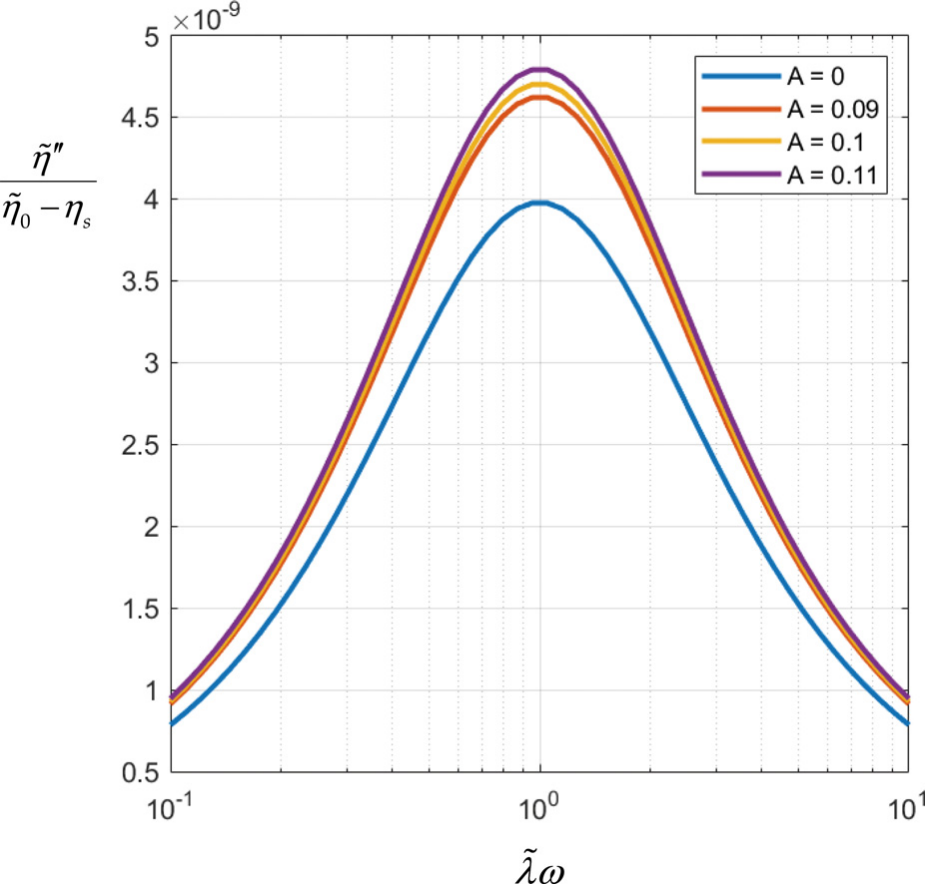
Dimensionless normalized $\eta^{\prime \prime }\left(\lambda \omega \right)$ from Eq. (2) parametrized with hydrodynamic interaction parameter, A, over the physical range arrived at for the coronavirus, Eq. (8).

From Fig. 4, we learn that over the physical range of the hydrodynamic interaction parameter, Eq. (8), the real part of the complex viscosity of the coronavirus suspension decreases with hydrodynamic interaction. Otherwise put, hydrodynamic interaction makes the coronavirus suspension more non-Newtonian. However, from Fig. 4 we also learn that the real part of the complex viscosity decreases only slightly with frequency (witness the ordinate gradation magnitude). From Fig. 5, we learn that over the range Eq. (8), *minus* the imaginary part of the complex viscosity of the coronavirus suspension increases with hydrodynamic interaction. Otherwise put, hydrodynamic interaction makes the coronavirus suspension more non-Newtonian in this sense too. However, from Fig. 5, we also learn that *minus* the imaginary part of the complex viscosity increases only slightly with frequency (witness the ordinate gradation magnitude). By non-Newtonian, we thus mean either the rise of *minus* the imaginary part or the fall of the real part of the complex viscosity.

Table IV summarizes our results over the peplomer biological hydrodynamic interaction range Eq. (8). We organize Table IV following previous works (Tables V–XIV of Refs. 15 and 16) and thus cast our results in dimensionless terms. From column 11 of Table IV, we learn that to the rotational diffusivity, hydrodynamic interaction matters. Specifically, over the peplomer biological hydrodynamic interaction range of Eq. (8), using Eq. (3) with column 7 of Table IV, we get

$$3.91\leq \lambda_{0}{\tilde{D}}_{r}\times 10^{4}\leq 4.05,$$ (9)

which falls well above the value without hydrodynamic interaction, $\lambda_{0}{\tilde{D}}_{r}=3.36\times 10^{4}$. Otherwise put, we find that hydrodynamic interaction of peplomers of the fully populated capsid increases its rotational diffusivity, and as the peplomer bulbs enlarge, the rotational diffusivity increases. Equation (9) and its companion Eq. (8) are the main results of this work.

**TABLE IV.** Coronavirus characteristics from general rigid bead-rod theory with hydrodynamic interaction.

A	$\frac{{\tilde{I}}_{1}}{\zeta mL^{2}}$	$\frac{{\tilde{I}}_{2}}{\zeta mL^{2}}$	$\frac{{\tilde{I}}_{3}}{\zeta mL^{2}}$	$\tilde{a}$	$\tilde{b}$	$\tilde{\nu }$	$\frac{2\tilde{b}}{\tilde{a}\tilde{\nu }}$	$\frac{{\tilde{\eta }}_{0}-\eta_{s}}{\mathrm{nkT}\tilde{\lambda }}$	$\frac{\tilde{\lambda }}{\lambda_{0}}$	$\lambda_{0}{\tilde{D}}_{r}$	$\frac{{\tilde{\Psi }}_{0,1}}{\tilde{\lambda }({\tilde{\eta }}_{0}-\eta_{s})}$
0	247.7566	247.7019	247.7915	123.8841	1.19 × 10^−8^	0.024 217	$7.96\times 10^{-9}$	1.500 071	495.5131	0.000 336	$1.59\times 10^{-8}$
0.09	213.2923	213.2216	213.2392	123.8841	1.61 × 10^−8^	0.028 130	$9.25\times 10^{-9}$	1.742 456	426.5846	0.000 391	$1.85\times 10^{-8}$
0.10	209.4880	209.4151	209.4243	123.8841	1.67 × 10^−8^	0.028 641	$9.41\times 10^{-9}$	1.774 098	418.9760	0.000 398	$1.88\times 10^{-8}$
0.11	205.6363	205.5611	205.5616	123.8841	1.73 × 10^−8^	0.029 178	$9.59\times 10^{-9}$	1.807 328	411.2726	0.000 405	$1.92\times 10^{-8}$

## IV. CONCLUSION

In this short paper, we explore the interactions between nearby peplomer bulbs (Sec. II). By interactions, we mean the hydrodynamic interferences between the velocity profiles caused by the drag of the suspending fluid around each peplomer when the virus rotates [defined by Eq. (6)]. We identify the primary hydrodynamic interactions with the edges of the 74-vertex polyhedron that is the solution to the Thomson problem (illustrated and animated in Fig. 2). We find that, for the well-known dimensions of the coronavirus [Eq. (7)], we get the physical range for the coronavirus hydrodynamic interactions. Said physical range, Eq. (8), is a main result of this work. We further find that coronavirus peplomer hydrodynamic interactions raise rotational diffusivity of the virus, and thus affect its ability to infect (column 11 of Table IV). We also find that over Eq. (8), the physical range of the hydrodynamic interaction parameter, A, the real part of the complex viscosity of the coronavirus suspension decreases with hydrodynamic interaction (Fig. 4). Otherwise put, hydrodynamic interaction makes the coronavirus suspension more non-Newtonian. Finally, we also learn that over the range Eq. (8), *minus* the imaginary part of the complex viscosity of the coronavirus, suspension increases with hydrodynamic interaction (Fig. 5). Otherwise put, hydrodynamic interaction makes the coronavirus suspension more non-Newtonian in this sense too.

In this work, we focus entirely on $N_{p}=74$, that is, on the fully populated virus at the time of infection. Our previous work on coronavirus rotational diffusivity without peplomer hydrodynamic interaction, $\lambda_{0}D_{r}$, spans the range $10\leq N_{p}\leq 100$ (see abscissa of Fig. 12 of Ref. 1). To incorporate peplomer hydrodynamic interaction over range $10\leq N_{p}\leq 100$ to get $\lambda_{0}{\tilde{D}}_{r}$ over the range of Eq. (8) would require a detailed analysis of the edges of each polyhedral solution to the Thomson problem (and the generation of a table akin to our Table III) for each $N_{p}$. This daunting task will require automation, and we leave it for another day.

We have restricted this work to spherical capsids, but we know coronavirus to be pleomorphic,^4^ and we know that this pleomorphism matters to its rotational diffusivity (see column 11 of Table III of Ref. 4). We leave the incorporation of peplomer hydrodynamic interaction in aspherical coronavirus suspensions for another day. For a dispersity of coronavirus in suspension incorporating peplomer hydrodynamic interaction, we would follow the well-known method of Problem 14C.2 of Ref. 17 (see also Sec. 26. of Ref. 18).

We have further restricted this work to the lowest energy peplomer arrangement,^19,20^ that is, the most likely polyhedral solution to the Thomson problem; however, we know of less likely solutions, and thus expect the $N_{p}=74$ population to be a dispersity of polyhedral arrangements. We leave the exploration of this dispersity (and the corresponding incorporation of its hydrodynamic interactions) for another day.

## Data Availability

The data that support the findings of this study are available within the article.
